# The impact of patient narratives on medical students’ perceptions of shared decision making: A randomized controlled trial

**DOI:** 10.1080/10872981.2021.1886642

**Published:** 2021-02-16

**Authors:** Marie Eggeling, Martina Bientzle, Simone Korger, Joachim Kimmerle

**Affiliations:** a Knowledge Construction Lab, Leibniz-Institut Fuer Wissensmedien, Tuebingen, Germany; b Department of Psychology, University of Tuebingen, Tuebingen, Germany

**Keywords:** Interprofessional collaboration, medical students, physiotherapy, learning, attitude

## Abstract

Successful shared decision making (SDM) in clinical practice requires that future clinicians learn to appreciate the value of patient participation as early as they can in their medical training. Narratives, such as patient testimonials, have been successfully used to support patients’ decision-making process. Previous research suggests that narratives may also be used for increasing clinicians’ empathy and responsiveness in medical consultations. However, so far, no studies have investigated the benefits of narratives for conveying the relevance of SDM to medical students. In this randomized controlled experiment, N = 167 medical students were put into a scenario where they prepared for medical consultation with a patient having Parkinson disease. After receiving general information, participants read either a narrative testimonial of a Parkinson patient or a fact-based information text. We measured their perceptions of SDM, their control preferences (i.e., their priorities as to who should make the decision), and the time they intended to spend for the consultation. Participants in the narrative patient testimonial condition referred more strongly to the patient as the one who should make decisions than participants who read the information text. Participants who read the patient narrative also considered SDM in situations with several equivalent treatment options to be more important than participants in the information text condition. There were no group differences regarding their control preferences. Participants who read the patient testimonial indicated that they would schedule more time for the consultation. These findings show that narratives can potentially be useful for imparting the relevance of SDM and patient-centered values to medical students. We discuss possible causes of this effect and implications for training and future research.

**Trial registration**: The study was pre-registered on the pre-registration platform *AsPredicted* (aspredicted.org) before data collection began (registration number: #29,342). Date of registration: 17 October 2019.

## Background

Shared decision making (SDM) has become increasingly common in medical consultations. SDM is generally important and of great significance in virtually all medical decisions, but this is especially the case in preference-sensitive situations[1]. Decision situations are preference-sensitive when different treatment options exist, and no option is deemed superior based on the available evidence. In these situations, personal preferences and values of the patients should be particularly taken into consideration in the treatment decision [2–4]. Using SDM has been found to positively influence patient participation[5], physician-patient relationship[6], and patient satisfaction [7–9], among other things. Most patients also wish to be involved in medical decisions which affect them [2,10,11].

Although SDM has many advantages, there are challenges regarding its implementation in clinical routine. Many clinicians feel that they lack the time for lengthy conversations[12], tend to overestimate patients’ understanding of medical information[13], and have difficulties explaining complex information in a comprehensible way [14,15]. Patients, on the other hand, often feel that they lack the knowledge to participate in treatment decisions and find it hard to understand the importance of their personal preferences[16]. Physicians often tend to give clear recommendations, and many patients expect them to do so [16,17]. Recommendations, however, are likely to influence the patients’ own decisions and possibly pull them away from their personal preferences [18–20].

The goal of this study was to explore ways to improve support for clinicians to develop a greater willingness to involve patients in the decision-making process. One possibility that has been considered and whose effectiveness is to be investigated is the use of patient narratives, which have been successfully applied in patient education so far and which we aim to transfer to medical education. We have chosen SDM for patients with idiopathic Parkinson disease (IPD) as a suitable test case because the decision between medication and surgical approaches for the treatment of IPD patients is a prime example of a preference-sensitive situation (see below for details). In the following presentation we first review the role of patient narratives in medical education. Then we discuss the significance of SDM for patients with IPD. Finally, we derive hypotheses from these considerations.

## Patient narratives in medical education

Narrative communication has been successfully applied in patient education, for example as a part of medical decision aids [21,22]. The use of narratives may be advantageous, because narratives provide information in a more vivid and understandable way than pure factual information. They are also more engaging, can help patients to imagine the consequences of a decision, and can help them to understand the emotions involved [23,24]. Their use is at the same time critically discussed, because narratives can be persuasive, which is problematic in preference-sensitive situations. However, more recent studies have found that this problem mainly occurs if the narrative focuses on the *outcome* of a treatment decision [24,25]. Just as patient narratives help other patients imagine what a treatment would be like, they can also be used for clinicians to demonstrate in a more vivid way that patients wish to participate in the decision-making process, and what such participation could look like. Narratives can also evoke empathy and new perspective. It has been found that medical experts’ empathy tends to decrease during their studies and first years of practice. This could originate from the expectation that they remain professional and maintain emotional distance during consultations[26]. This may be problematic, however, because former research suggests that empathy of physicians can be a means to improving patient-physician relationships [27,28] and that physicians’ ability to take the patients’ perspective is correlated with higher patient satisfaction[29].

The use of training programs that help health professionals prepare for SDM has become more common[30]. Making the effort to hear patient perspectives was perceived positively by medical students[31] and led to increased confidence when consulting patients from marginalized groups[32]. However, these studies were not randomized trials and lacked control groups. Also, many of the training programs were lengthy, which is likely to be a barrier when implementing them in practice. The question arises whether just making interventions shorter could have a positive effect on health professionals’ ability and motivation to engage in SDM. A randomized controlled trial with medical undergraduates compared the effects of their viewing a patient experience video with a doctor video[33]. It was found that viewing the patient experience not only led to stronger feelings of comfort and confidence in communication skills, but also to better performance in clinical exams. These are promising results. In the study presented here, we investigated in a randomized controlled trial whether reading a short narrative about the SDM experience of a patient improved medical students’ attitudes toward and their motivation to engage in SDM. We selected a consultation of an IPD patient as a test case for the impact of narratives on SDM.

## SDM for patients with Parkinson disease

Previously, medication was the only available treatment in the early stage of IPD. But over time, surgical approaches, such as deep brain stimulation (DBS), have developed as potential treatment options, when symptoms cannot be treated sufficiently with medication[34], or when medication causes massive side effects [35,36]. In 2016, the Association of Scientific Medical Societies in Germany and the German Society of Neurology[35] recommended offering DBS to younger patients in an early stage of the disease, or to patients whose symptoms cannot be controlled with medication alone anymore. But it is also emphasized that surgical treatment remains an individual decision as long as medication alternatives still exist, especially because surgery is always associated with certain risks which individuals may want to avoid [35,37]. As IPD is a chronic disease, it cannot be cured, and both pharmaceutical and surgical approaches (DBS) can be applied only to improve the symptoms and in turn the quality of life. Patients may have different priorities about what is important to them, making a case for SDM[35]. Current studies with IPD patients show that many prefer to take the lead or be included in the treatment decision[38].

IPD is a slowly progressing, neurodegenerative disease, and consequently the decision-making process regarding treatment can take a relatively long period of time [39]. In order to support patients during this time, physicians first need to know about the alternative treatment options themselves, and second, they need to understand the relevance of SDM in this situation. Previous research shows that many neurologists do not consider DBS as a possible treatment option for patients with IPD. Hamberg and colleagues [40–42] reported that patients have to ‘struggle to convince their clinicians to refer them for an assessment by a DBS team’ (p. 1). Apparently, neither neurologists nor patients are sufficiently informed about DBS [28–30]. For instance, neurologists overestimate the risks of the surgery and underestimate patients’ readiness to consider DBS as a treatment option. Consequently, there is an urgent need to inform neurologists that DBS may be a promising treatment option for some IPD patients.

But even if physicians are better informed about DBS and offer it to patients as an option, they still need to consider the patients’ personal preferences regarding this decision and provide medical information about the treatment options. So, physicians should also be educated about the importance of SDM in this situation and about ways to implement it in practice. The goal of this study was to educate medical students about both the treatment options and the relevance of SDM.

## Hypotheses

We aimed to test whether there is an impact of presentation style on participants’ perceptions of SDM. We hypothesized that participants who read a narrative patient testimonial would perceive SDM to be more important than participants who read a purely fact-based information text (Hypothesis 1).

We also aimed to test whether there is an impact of presentation style on participants’ control preferences (i.e., their priorities as to who should make the decision). We hypothesized that preferences for a collaborative role in the SDM process would be stronger for those participants who read a narrative patient testimonial than for participants who read a fact-based information text (Hypothesis 2).

As an exploratory question, we investigated the impact of presentation style on the time participants intended to schedule for a consultation.

## Methods

The study adhered to CONSORT guidelines and was designed as an online experiment with presentation style (narrative patient testimonial vs. fact-based information text) as between-group factor. Perceptions of SDM, control preferences, and intended consultation time served as dependent variables. The study was pre-registered on the pre-registration platform *AsPredicted* (aspredicted.org) before data collection began (registration number: #29,342).

## Participants

The intended sample size of N = 180 was determined by power analyses for t-tests (N = 176) and Mann-Whitney-U (N = 184) test with α = .05, an intended power of 95%, and a medium effect size of d = 0.5. The participants were recruited via a mailing list of the online learning platform Sectio Chirurgica [43–45], an online video platform for medical students and health professionals. Any medical student who was 18 years or older and spoke German could participate in the study.

## Intervention

After responding to demographic questions, participants were put into a hypothetical scenario where they prepared for a consultation with an IPD patient. In order to inform them about the treatment options, they watched a video containing general information on IPD and two common treatments (medication and DBS). The video (3:17 min) was edited for this study based on a video from the Sectio Chirurgica platform about DBS. Afterwards, participants were randomized by a computer program to read either a narrative patient testimonial or a purely fact-based information text (see Appendix A). Participants were blinded to the intervention. Then we captured their perceptions of SDM, control preferences, and the intended consultation time. Finally, the participants were debriefed.

## Measurements

*Perceptions of SDM* were measured based on the survey of King and colleagues[46]. It contained questions about 1) perceived importance of SDM in different situations, 2) attitude toward participation of IPD patients, and 3) attitude toward SDM in situations where more than one treatment option exists (see Table 1).

**Table 1.** Perceptions of SDM

	Very unimportant			Very important	
*Perceived importance of SDM*					
In your own opinion, how important is a shared decision-making process in making medical decisions in the following medical situations?					
Changing health behavior		▢ ▢	▢ ▢ ▢	▢ ▢	
Taking medication		▢ ▢	▢ ▢ ▢	▢ ▢	
Surgical interventions		▢ ▢	▢ ▢ ▢	▢ ▢	
Coping with chronic diseases		▢ ▢	▢ ▢ ▢	▢ ▢	
	Only the patient	Mostly the patient	Both patient and clinician equally	Mostly the physician	Only the physician
*Attitude toward participation of IPD patients*					
When you think about treatment decisions for Parkinson disease, who do you think…					
… should make decisions?	▢	▢	▢	▢	▢
… actually makes decisions?	▢	▢	▢	▢	▢
	Not important at all			Very important	
*Attitude toward SDM in situations where more than one treatment option exists*					
In cases where there is more than one treatment option, how important do you think the shared decision-making process is to patients?		▢ ▢	▢ ▢ ▢	▢ ▢	

*Control preferences* were measured with the scale of Degner and colleagues[31] (see Table 2).

**Table 2.** Control preferences

The following statements describe different roles that a physician can have in medical decision situations. Please sort the statements into your preferred order. This means the role you as physician would most like to play in the first position and the role you would least like to play in the last position.	
▢	My patient should make the decision alone. (1)
▢	My patient should make the decision, but take my opinion into account. (2)
▢	My patient and I should make the decision together. (3)
▢	In the end I should make the decision, but I should seriously consider my patient’s opinion beforehand. (4)
▢	I should make the decision alone. (5)

As an exploratory measure, participants were asked how much time they would schedule for the consultation. For this purpose, they indicated the minutes as integers.

## Analysis

Data analysis was performed using IBM SPSS 25 for Windows. To test for differences between the conditions, we performed t-tests (for interval-scaled data) and a Mann-Whitney test (for ordinal-scaled data). The data are reported as means (M) ± standard deviations (SD) for interval-scaled data and the median for ordinal-scaled data. The level of significance was set at p < 0.05. Cohen’s d was calculated as effect size with d > 0.35 demonstrating a meaningful effect size.

## Results

**Figure 1.**
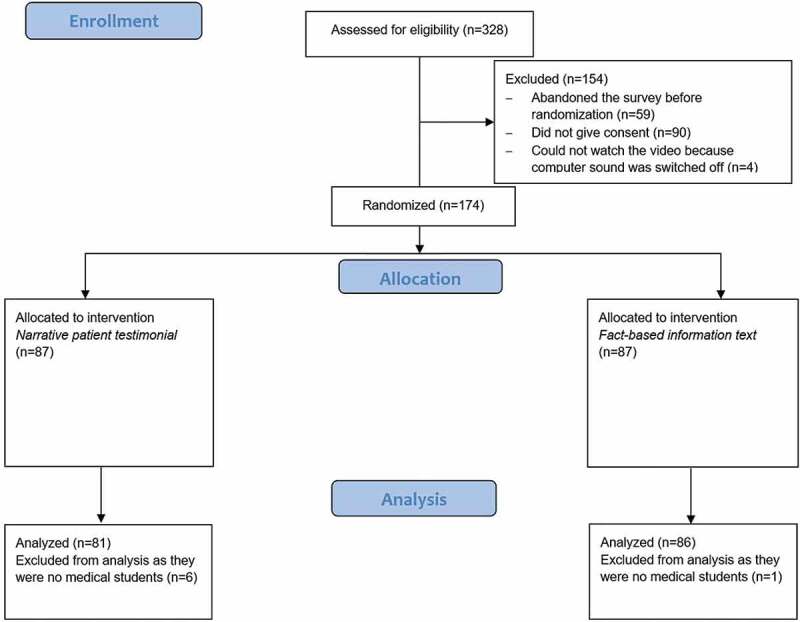
Flow diagram of study design

N = 167 medical students (age: M = 24.47 years, SD = 3.60 years; gender: 96 female, 70 male, 1 diverse) were included in the analysis. Most participants (n = 163) were already in the clinical phase of their studies (duration of studies: M = 7.51 semesters, SD = 2.16 semesters). The complete participant flow is presented in Figure 1.

## Equivalence of student groups

Before analyzing treatment effects, several variables were checked for equivalence among the conditions. They did not differ from each other regarding age (p = 0.938), gender (p = 0.263), or duration of studies (p = 0.344).

## Perceptions of shared decision-making

The internal consistency of the *perceived importance of SDM* scale was poor (Cronbach’s alpha = .68). Due to this psychometric shortcoming, no analyses of this scale could be conducted.

The analysis of the *attitude toward participation of IPD patients* showed that participants in the narrative condition (M = 2.60, SD = 0.61) more strongly indicated the patient as the one who should make decisions than participants in the information text condition (M = 2.87, SD = 0.61; t_(165)_ = 3.70, p = 0.005, d = 0.44). Participants in the narrative condition believed that IPD patients should be more involved in the decision than the physician (test against the mean value of the scale [3]: t_(80)_ = −5.87, p < 0.001, d = 0.66). This was not the case for participants of the information text condition (t_(85)_ = −1.95, p = 0.055). Regarding the rating of who actually makes the decisions, the conditions did not differ from each other (p = 0.173). Both groups tended to believe that it is the physician who really makes decisions rather than the patient (M = 3.62, SD = 0.79; test against the mean value of the scale [3]: t_(165)_ = 10.10, p < 0.001, d = 0.78).

The findings regarding the *attitude toward SDM in situations where more than one treatment option exists* were in line with Hypothesis 1. Participants who read the patient narrative considered SDM to be more important (M = 6.35, SD = 0.90) than participants who read the information text (M = 6.01, SD = 0.95; t_(165)_ = 2.33, p = 0.021, d = 0.37).

## Control preferences

Contrary to Hypothesis 2, there were no significant group differences in control preferences. In both conditions, the participants as physicians preferred to make the decision together with the patient (Median_narr_ = 3, Median_fact_ = 3; U = 3439.00, p = 0.865).

## Exploratory analysis – intended conversation time

Participants in the narrative condition scheduled significantly more time for the consultation (M = 20.54 min, SD = 6.58 min) than participants of the factual information condition (M = 17.68 min, SD = 5.50 min; t_(164)_ = 3.04, p = 0.003, d = 0.47).

DISCUSSION

The goal of this study was to illuminate the impact of an intervention with short, text-based patient narratives on medical students’ perceptions of SDM. In this randomized controlled trial, we found that medical students who read a patient testimonial perceived SDM in situations where more than one treatment option exists as more important than participants who read an information text. We also found that medical students in the narrative condition rated patient participation as more relevant than participants in the information text condition. This positive perception of SDM may also be reflected in the longer period of time that medical students in the narrative condition scheduled for the medical consultation – although it is speculative at this point whether the greater amount of time participants were willing to invest was actually an indicator of a more positive perception of SDM; there may be other reasons for this finding. Nevertheless, this is a highly relevant finding, as time constraints are often mentioned as a key barrier to SDM[16]. The willingness of future doctors to invest in more consultation time is particularly important against the background that ‘(c)hanging attitudes alone will not create time for shared decision making’ [47,48] (p. E2). Our findings suggest that both the attitude and the intention to take more time may go hand in hand. Regarding the role the participants would like to play as physicians in a medical decision process, however, there were no significant group differences. Making the decision together with the patient was the most favored approach for medical students in both conditions.

The participants in this study were medical students in an advanced stage of their education and, overall, their perception of SDM was quite positive. This was reflected in this sample in their control preferences. Physician-patient communication and SDM are included in the national competence-based catalogues of learning objectives for undergraduate medical education in Germany^49^. Thus, it is likely that the participants had already learned basic principles of SDM during their studies or at least had known what course of action was desired. Therefore, we cannot rule out that patient narratives could still have a future impact on medial students’ control preferences.

## Limitations and further steps

This study has some limitations. We did not reach the intended sample size of N = 180. This means that we cannot interpret non-significant results as non-existent effects. It is possible that our test power was not sufficient to find the effects. 328 participants were assessed for eligibility, but 154 were excluded already before randomization. Ninety participants just clicked on the e-mail link and did not give consent for participation, another 59 participants abandoned the study before randomization. This entails the risk of having a selective sample that, for example, has shown more interest in the topic than other medical students.

Moreover, we did not measure real SDM behavior in a clinical setting. Our measurements of the perception of SDM and the intention to schedule enough time for a medical consultation were preliminary to actual application. However, a positive attitude toward SDM is indeed supposed to increase the probability that people will engage in actual SDM behavior. A positive relationship between attitudes and behavior can typically be found, especially for strong attitudes (for an overview see^50^). The strength of this relationship should be investigated in future research in clinical contexts.

We have used three different measures of SDM perception. Another limitation is that the adaptation of the subscale *Perceived importance of SDM* to the current study resulted in a low reliability. In addition, the generalizability of the findings is limited, as only medical students took part in the study. We therefore do not know whether the intervention would also be suitable for other medical experts and health professionals.

A further limitation is that we did not use a pre-post-design. We can therefore make no conclusion about the impact of our intervention on the modification of our dependent variables. But since we have used a randomized study design the differences between the conditions can most likely be attributed to the intervention.

## Data Availability

Data are available on request to Martina Bientzle.

## Declarations

**Ethics approval and consent to participate**

This research was performed in accordance with the Declaration of Helsinki. The study had full approval by the ethics committee of the Leibniz-Institut für Wissensmedien (approval number: LEK 2019/037). All participants took part voluntarily and anonymously. They gave written informed consent and were informed about privacy protection, their right to terminate participation at any time without any disadvantage, and about the general purpose of the study.

## Narrative patient testimonial

When I was first diagnosed with Parkinson’s disease, I was naturally shocked. I knew that there was no cure for Parkinson’s and that later on the whole body would be affected. The idea scared me very much, especially because my grandfather was also suffering from Parkinson’s and I could remember how badly he often felt. The doctor encouraged me and explained to me that the treatment options for Parkinson’s patients had improved significantly over the last decades and that I should not compare my situation with my grandfather’s.

Over time, I have learned to deal with the disease and realized that I can do a lot. I go to a self-help group and the exchange with the others does me a lot of good. I also go to physiotherapy, which is also good for me because I realize that I can still do something physically despite the illness. Fortunately, I get a lot of support from my friends and acquaintances, so that I can deal with the illness openly.

Nevertheless, it is difficult to deal with the disease. When my symptoms are stronger, especially the tremor, I often don’t feel like going anywhere. I am afraid to isolate myself further and further, although I have always been a sociable person. I know that many patients with Parkinson’s disease feel similar. Although I have actually had some experience with the disease, I am still afraid that I am no longer in complete control of my body.

I was first treated with a very low-dose medication. It helped very well against my symptoms, but it caused me problems sleeping. I was often tired and in a bad mood during the day. When I dared to talk to my doctor about this, he tried to adjust the treatment to my individual needs and prescribed a different medication. He encouraged me to bring in my experience with the medication and my wishes regarding the treatment.

Because I know that the disease is getting worse and worse, I have also thought about other treatment options. I have heard that deep brain stimulation works well for many patients. Most of them do it when there is no other way, but I have read that it is also a possibility at an earlier stage of the disease - as it is for me. That’s why I would like to talk to my doctor about how this looks like in my case.

I am very unsure whether such a surgery would be the right thing for me. The thought of having my head drilled and something put into my brain is pretty scary and I also have the worry that I won’t really be myself after that, because sometimes there are changes in feelings or personality. Supposedly such changes are reversible, but it is still not a pleasant thought. I’m also afraid of complications - this only happens to a few patients, but what if I were one of them? On the other hand, I am aware that my disease will get worse over time and that at some point I may have no choice but to do deep brain stimulation. I already realize that the dosage of the medication has to be increased again and again and that I still notice some symptoms clearly. I know that the effect of the drugs may diminish, so that I may soon have to take additional, stronger drugs. With deep brain stimulation, the dosage could be reduced again for the time being. However, I have heard that deep brain stimulation can also have a habituation effect, whereupon the intensity of the stimulation must be increased.

I would like to have more motivation to go to events or do real sports. In the past I did a lot of sports and was very active, but due to the illness this has already decreased considerably. It is important to me that I can still lead a normal and independent life as long as possible and I would like the best possible treatment for this.

## Fact-based information text

Parkinson’s disease or Morbus Parkinson (further synonyms: Idiopathic Parkinson’s syndrome, colloquially also called shaking disease) is a slowly progressive loss of nerve cells. As an incurable neurodegenerative disease, it is one of the degenerative diseases of the extrapyramidal motor system. Approximately one percent of the world’s population over the age of 60 is affected by this disease. Parkinson’s disease is thus the second most common neurodegenerative disease in the world.

Typical symptoms are muscle tremor, muscle rigidity, slowed movements that can lead to immobility, postural instability and ‘out-of-round’ movements when running. In addition, sleep disorders, digestive problems, disorders of the sense of smell and mood swings often occur.

The disease begins insidiously and then progresses throughout the patient’s life, the symptoms become stronger in the course of the disease and are therefore easier to recognize. An early sign is, for example, the reduced and later missing swinging of an arm while walking. It is not uncommon for shoulder pain and one-sided muscle tension to occur, which first leads the patient to an orthopedic specialist.

Today, there is still no possibility of a causal treatment of Parkinson’s syndrome, which would consist in preventing or at least halting the progressive degeneration of the nerve cells of the nigrostriatal system. Therefore, treatment of the symptoms must be satisfied with a treatment that is increasingly feasible, allowing patients to live an almost unhindered life, at least in the first years (sometimes even decades) of the disease.

Treatment is mainly through the administration of a dopaminergic medication, i.e., drugs that increase the supply of dopamine in the brain or drugs that replace the missing dopamine. The most important drug is L-dopa (levodopa), a precursor of dopamine.

Due to the drugs, the symptoms decrease significantly, especially in the early stages of the disease. As the disease progresses, treatment becomes more difficult. In addition, sometimes stressful side effects can occur (e.g., sleep disorders). It is difficult to predict how successful the treatment will be. The drugs do not work in the same way for every person - and sometimes it takes time to find the right dose. A noticeable effect usually sets in within one to two weeks after the start of treatment: Movements become easier again and stiffness decreases. Such problems can be further reduced up to three months after the start of treatment. The tremor is often more difficult to treat. Sometimes it only disappears after months or even years of drug treatment.

For many years, neurosurgical treatment options have also been used for Parkinson’s disease. One method used since the early 1990s is deep brain stimulation (DBS), in which a programmable pulse generator is applied to the patient. It generates electrical impulses and conducts them via thin cables, depending on the localization of the cause of the disease and the corresponding placement of the stimulation electrodes, into the respective basal ganglia subthalamic nucleus, globus pallidus, or the anterior thalamus, whereby overactive false impulses can be effectively suppressed there.

The electrode placement procedure is a stereotactic brain operation that takes about six to twelve hours and requires precise planning and control based on radiologically acquired spatial image data and electrically derived neurophysiological measurements both before and during the operation.

The effect of the surgery is long lasting. However, Parkinson’s disease also progresses with stimulation, so this treatment is not a cure.

DBS is usually only considered in Parkinson’s patients after they have been ill for many years and no further improvement can be achieved with medication. However, studies suggest that it may be useful to operate on patients in earlier stages. Figure 1 - Flow diagram of study design
